# Supplementary material for: Nd3+, Yb3+:YF3 Optical Temperature Nanosensors Operating in the Biological Windows
Source: Materials (Basel). 2022 Dec 21;16(1):39. doi: 10.3390/ma16010039 (PMC9821644; doi:10.3390/ma16010039)
Supplement: Supplementary file 1 [file materials-16-00039-s001.zip › materials-2030604-supplementary.pdf]

materials

MDPI

Supplementary information

Nd^{3+}, Yb^{3+}:YF_3 optical temperature nanosensors operating in the biological windows

Maksim Pudovkin*, Ekaterina Oleynikova, Airat Kiiamov, Mikhail Cherosov and Marat Gafurov*

18th Kremlyovskaya street. Kazan 420008, Russian Federation, Institute of physics
Correspondence: M.P., jaz7778@list.ru and M.G.**, mgafurov@gmail.com

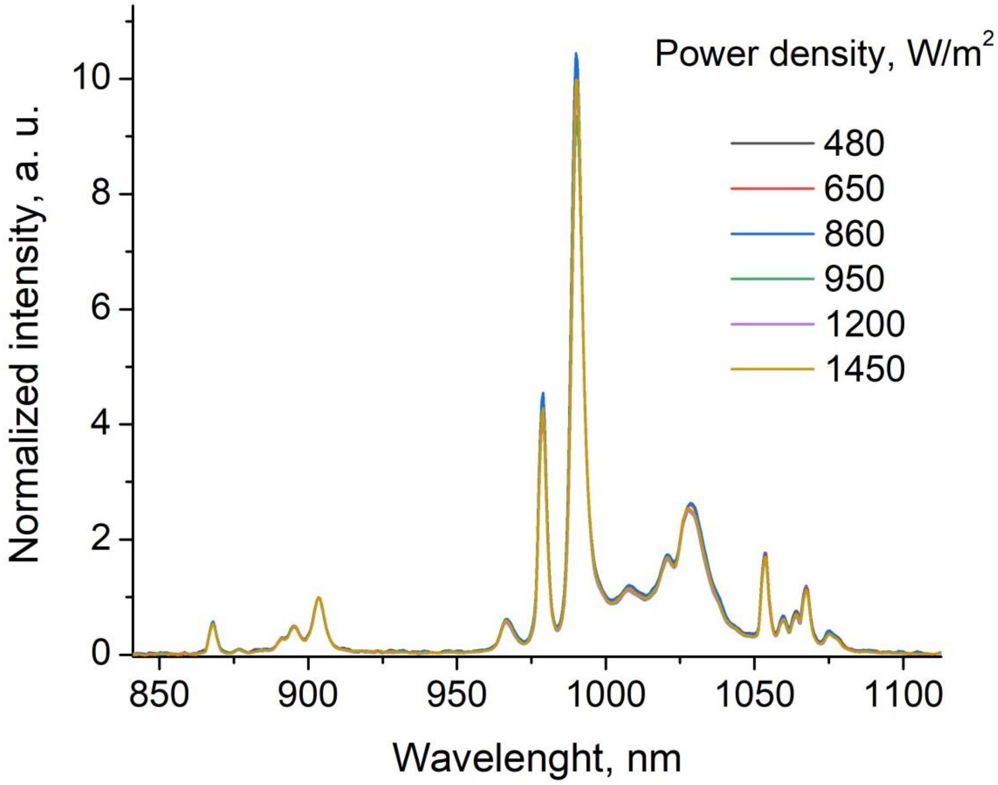

Figure S1. The luminescence spectra recorded at different values of power density of the laser irradiation. It can be seen, that the shape of the spectra are independent of the power density. It can be related to the lack of heating. We used the maximum power values aroung 300 mV that corresponmds to ~ 1300 W/m2. These values are comparable to our previous work “https://doi.org/10.1016/j.optmat.2021.111328”.

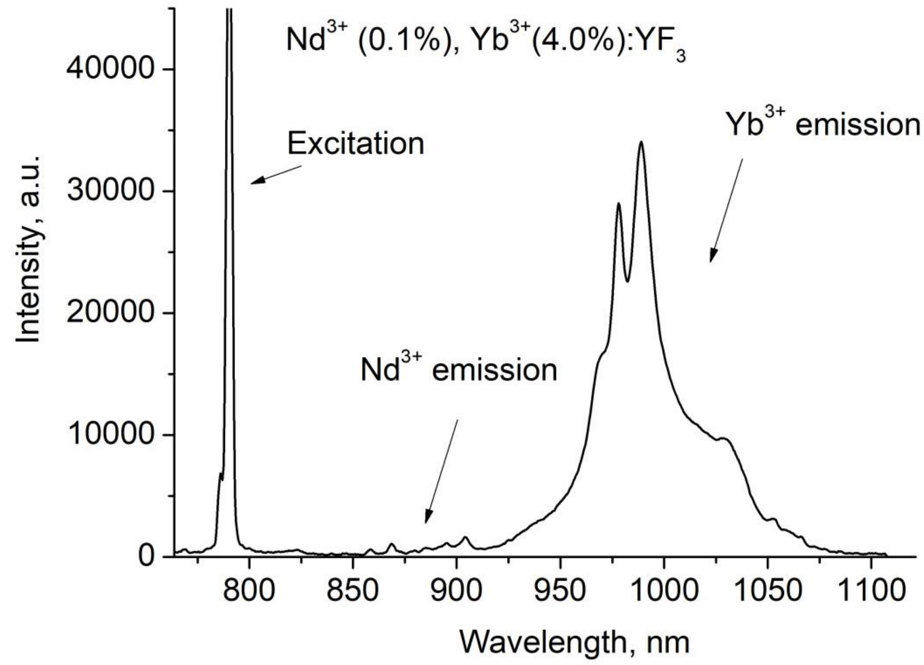

Figure S2. Room temperature luminescence spectrum of Nd^{3+}(0.1%), Yb^{3+}(3.0%):YF_3. It can be seen, that the integtated intensity of Yb^{3+} much higher compared to Nd^{3+} one. This pecularity leads to deterioration of the performances.
